# Cognitive change trajectories in virally suppressed HIV-infected individuals indicate high prevalence of disease activity

**DOI:** 10.1371/journal.pone.0171887

**Published:** 2017-03-06

**Authors:** Chloe Gott, Thomas Gates, Nadene Dermody, Bruce J. Brew, Lucette A. Cysique

**Affiliations:** 1 Psychology Department, Macquarie University, Sydney, NSW, Australia; 2 Faculty of Medicine, University of New South Wales, Sydney, NSW, Australia; 3 Departments of HIV and Neurology St Vincent’s Hospital and Peter Duncan Neurosciences Unit St Vincent’s Centre for Applied Medical Research Centre, Darlinghurst, NSW, Australia; 4 Neuroscience Research Australia (NeuRA), Sydney, NSW, Australia; Nathan S Kline Institute, UNITED STATES

## Abstract

**Background:**

The longitudinal rate and profile of cognitive decline in persons with stable, treated, and virally suppressed HIV infection is not established. To address this question, the current study quantifies the rate of cognitive decline in a cohort of virally suppressed HIV+ persons using clinically relevant definitions of decline, and determine cognitive trajectories taking into account historical and baseline HAND status.

**Methods:**

Ninety-six HIV+ (clinically stable and virally undetectable) and 44 demographically comparable HIV- participants underwent standard neuropsychological testing at baseline and 18-months follow-up. We described clinically relevant cognitive trajectories based on standard definitions of historical and baseline HAND status and cognitive decline. Historical, moderate to severe HAND was formally diagnosed at the start of the cART era in 15/96 participants based on clinical neurological and neuropsychological assessment. The same standard of care has been applied to all participants at St. Vincent’s Hospital Infectious Disease Department for the duration of their HIV infection (median of 20 years).

**Results:**

Relative to HIV- controls (4.5%), 14% of HIV+ participants declined (*p* = .11), they also scored significantly lower on the global change score (*p* = .03), processing speed (*p* = .02), and mental flexibility/inhibition (*p* = .02) domains. Having HAND at baseline significantly predicted cognitive decline at follow up (*p =* .005). We determined seven clinically relevant cognitive trajectories taking into account whether participant has a history of HAND prior to study entry (yes/no); their results on the baseline assessment (baseline impairment: yes/no) and their results on the 18-month follow up (decline or stable) which in order of prevalence were: 1) No HAND history, no baseline impairment, 18-month follow-up stable (39%), 2) No HAND history, baseline impairment, 18-month follow-up stable (35%), 3) History of HAND; baseline impairment, 18-month follow-up stable (9%) 4) No history of HAND, baseline impairment, 18-month follow-up decline (7%), 5) History of HAND, no baseline impairment, 18-month follow-up stable (3%), 6) No HAND history, no baseline impairment, 18-month follow-up decline (3%) 7) History of HAND, baseline impairment, 18-month follow-up decline (3%). There was no relationship between cognitive decline (taking into account historical and baseline HAND) and traditional HIV disease biomarkers.

**Conclusions:**

Despite long-term viral suppression, we found mostly subclinical levels of decline in psychomotor speed and executive functioning (mental flexibility and cognitive inhibition); well-established markers of HAND progression. Moreover, 57% of our cohort is undergoing slow evolution of their disease, challenging the notion of prevalent neurocognitive stability in virally suppressed HIV infection.

## Introduction

Similar to the United Kingdom, as well as subgroups in the United States, Europe and Asia, the HIV epidemic in Australia is well controlled (with 92% of treated HIV-infected (HIV+) persons on combined anti-retroviral therapy (cART) virally undetectable)[1]. This context provides an optimal setting for the natural study of cognitive trajectories in chronic HIV infection.

The cross-sectional profile of HIV-associated neurocognitive disorder (HAND) is well established. It is described as three progressively more severe diagnoses; asymptomatic neurocognitive impairment (ANI), mild neurocognitive disorder (MND) and HIV-associated dementia (HAD [2]. In the context of stable cART, ANI and MND are the more prevalent sub-diagnoses occurring in 50%-80% and 10–20% of HAND cases respectively [3]; while HAD is rare (2%-4% of cases). HAND is characterized by a diffuse pattern of cognitive impairment consistent with disruption to the fronto-striatal- parietal networks. The most frequent deficits are to processing speed, which typically remains prominent across all severity stages of the disorder[4]. Episodic memory impairment is also prevalent [3] as are disruptions to complex attentional processes and executive functioning [4–6]. Motor signs are relatively uncommon in the milder stages and their onset is frequently reflective of disease progression[3]. While the cross-sectional profile of cognitive impairment is well described, the rate and profile of cognitive decline in persons with stable and treated HIV infection is far from established.

Indeed, there has been a lack of research examining longitudinal neuropsychological changes *in individuals with long-term viral suppression*, in whom HAND is mostly characterized by ANI and MND stages. Moreover, when cognitive decline has been investigated in these samples, varying methods of defining decline have been used. Some of which have had low clinical relevance and inadequate corrections for practice effect, potentially leading to erroneous estimates of cognitive decline.

We provide a summary of the nine naturalistic longitudinal studies of cognitive change that have been conducted in samples of HIV+ participants; which the majority of the sample were on stable cART and *cognitive decline* in the HIV+ group was investigated as a primary outcome (S1 Table). These nine observational studies represent the total pool of research as of July 2016 to the best of our knowledge. The following main findings can be extracted from this review: 1. The definition of cognitive decline as an absorbing state (wherein once an individual is defined as having declined this status is permanent) as compared to *studies with multiple follow-up points* (wherein an individual’s status can fluctuate across the duration of the project) yields *apparent* differences in cognitive decline rates that have to be interpreted carefully. Cysique and colleagues [7] defined cognitive decline as a unique endpoint and found 30% of HIV+ participants were defined as declined at 6–12 month follow-up and 5% at 15–27 months. In contrast, the large CNS HIV Antiretroviral Therapy Effects Research (CHARTER) study which defined cognitive decline as an absorbing state found an overall 23% cognitive decline rate over the three-year study follow-up [8]. A subsequent project also by Cysique and colleagues [9] also used an absorbing rate definition and found an overall decline over the 27 month study period of 21%. 2. Studies that used standard global change scores (*standard* in the sense that scores are corrected for practice effect and regression towards the mean) found fairly similar rates of cognitive decline ranging between 21%-27% across different cohorts context/nationalities [9–12]. 3. When focusing on deterioration within the HAND stages (thus not correcting for practice effect and regression towards the mean) lower rate of cognitive decline are typically observed. For example, The Multicenter AIDS Cohort Study (MACS) found that across four years of follow-up, only 10% of HIV+ individuals progressed to a worse HAND stage [13]. 4. In contrast, a study that concentrated on incident cognitive decline in 146 neurocognitively normal HIV+ participants and used a cognitively stable individuals as reference (thus correcting for both practice effect and regression towards the mean within the entire sample), detected 15% had incident cognitive decline 14.3 ± .2 months later [14]. 5. Studies that used a restricted number of tests showed inconsistent results. When focusing on verbal memory, one study found worse performance over time in HIV+ individuals compared to HIV-individuals at one year follow-up [15], while another which focused only on two tasks of psychomotor speed and mental flexibility reported stable cognitive performance across eight years [16]. 6. Finally, none of the above studies took into account the impact of individuals included in the study who may have previously been diagnosed with an HAND episode in the lifetime of their illness, especially before cART was available. Typically these individuals once put on cART have dramatically improved [17] Their long-term prognosis has however not been studied, which is important as historical HAND represents a risk for cognitive deterioration years after an initial episode [9, 18].

The aim of the current study was threefold; 1) to quantify the rate of incident neurocognitive decline in chronic treated HIV+ persons relative to healthy controls of the same age over an 18-month period, based on a test battery that assessed seven cognitive domains 2) To investigate the historical and baseline cognitive determinants of cognitive decline and to determine the profile of cognitive trajectories in functions of historical and baseline HAND status. 3). To determine whether standard HIV disease biomarkers were related to cognitive decline.

## Materials and methods

### Participants

The current investigation is based on an Australian middle-aged clinically stable and virally suppressed HIV+ cohort, for which the study details and procedures have already been published[19–21]. This cohort is composed of a majority of men having sex with men (MSM) with a high level of premorbid cognitive functioning.

One hundred and two HIV+ participants were recruited through the HIV and Neurology clinics at St Vincent’s Hospital between 2009 and 2011. Inclusion criteria for the project required HIV+ participants to i) be greater than 45 years old ii) be stable on cART for more than 6 months, iii) have a nadir CD4 equal or less than 350/μL and iv) have been HIV+ for more than 5 years. Fifty HIV-negative (HIV-) participants were recruited through advertising in metropolitan Sydney, at Holdsworth House Medical Practice, St Vincent’s Hospital and UNSW campuses from 2009 to 2013. Inclusion criteria for the HIV- group required participants i) to be over 45 years and to be ii) HIV- on an enzyme-linked immunosorbent assay (ELISA) test within the past three months at both baseline and follow-up. Exclusion criteria for both groups included i) a history of a neurological disorder (with the exception of HAND) ii) a psychiatric disorder on the psychotic axis iii) any current substance or alcohol use disorder iv) loss of consciousness (LOC) for greater than 30 minutes v) active Hepatitis C vi) or poor proficiency in English. Recruitment sites for both HIV+ and HIV- groups were in the same geographical area to ensure similar lifestyle and demographic factors in both groups. Ninety-six HIV+ (6% attrition) and 44 HIV- (12% attrition) participants returned for follow-up assessment designed to be at approximately 18 months (mean test-retest interval = 19.56 months, SD = 7.73 months) post baseline assessments. Two HIV- participants were excluded at follow-up: one with high levels of psychological stress related to a severely unwell partner and the other with chronic fatigue as a result of long-term difficulties with insomnia. On most illness and demographic variables there was no significant difference between participants retained and those lost to follow-up.

### Ethics

This project was approved by the Human Research Ethics Committees (HREC) of The University of New South Wales (UNSW), St. Vincent’s Hospital and Macquarie University. Individuals involved in the study provided informed written consent prior to participation at both baseline and follow-up.

### Procedure

Study visits at baseline and at 18 months were comprised of a standard medical and demographic history and an assessment of neurocognitive functioning. All neuropsychological measures were administered and scored according to standard procedures. The test battery covered seven cognitive domains previously shown to be sensitive to neurocognitive deficits that occur in HIV (S2 Table)[22–24]. At follow-up, where available, alternate versions of test measures were used (i.e. verbal learning/memory and letter fluency) to minimize practice effects.

### Historical HAND status definition

History of HAND was based on standard neurological and neuropsychological examinations, MRI/MRS scans and an extended panel of plasma and CSF biomarkers, as described previously [25]. LC consulted the medical records and recorded HAND diagnoses reported using the AIDS Dementia Complex nomenclature [26]. This was adapted to the HAND 2007 criteria [2] by LC, and reviewed by BJB (HIV Neurologist) to reach diagnoses of MND (%) and HAD (%) [2]. Past HAND status was analysed as a dichotomous variable (yes/no). Importantly, the majority of cases diagnosed with MND or HAD were diagnosed at the start of the cART era (median year = 2001, earliest year = 1996 and latest year = 2008). For most cases, MND (53%) and HAD (47%) was diagnosed in the context of treatment failure and/or drug resistance on the less optimal regimen at the start of the cART era. In a few cases, MND and HAD was diagnosed while untreated, because the patient was not aware of their HIV diagnosis or they presented late at the HIV Neurology clinics. All had advanced HAND with at least moderate cognitive and functional deficits. The clinical-research axis at St. Vincent’s hospital is represented in this cohort, with all cases maintained on the same standard of care for the duration of their HIV infection (median of 20 years). This means that while the non-historical HAND cases did not complete standard clinical neuropsychological testing, at no time in the history of their illness did they experience functional deficits, cognitive symptoms or neuropsychiatric symptoms (of same magnitude as the diagnosed cases) that would have triggered a referral to the neurologist (BJB). Historical HAND cases, all presented with advanced cognitive and functional deficits. Therefore cases with potentially milder form of the disease (i.e., ANI) were not identified through this clinical framework. However, all cases were identified before the advent of the Frascati criteria in 2007 (only one was diagnosed in 2008).

### Baseline HAND status definition

Baseline impairment status was determined using local normative standards (scaled scores) developed in a demographically comparable HIV- sample recruited as part of the HIV and Brain Aging research program at the University of New South Wales (PI, LAC). Details on methods and the sample characteristics used to develop local norms have been published [21]. The standard Global Deficit Score (GDS) method [27,28] was used to classify impairment. As per convention [27,28], GDS≥0.5 was used to define a clinically relevant level of impairment yielding a discrete outcome (impaired or unimpaired) or a continuous outcome. A higher GDS indicates greater impairment. The standard GDS cut-off of ≥0.5 is widely used to assess HIV-related brain injury and meets the international criteria for HAND [28]. For each participant, we determined the HAND classification as follows [2]: GDS≥ 0.5 & no Independence in Activities of Daily Living (IADL) decline = ANI; GDS≥ 0.5 & mild/moderate IADL decline = MND; GDS≥ 1.5 & severe IADL decline = HAD. IADL information was obtained from the Patients Assessment of Own Functioning Inventory [29] and any clinical evidence of IADL decline (medical records; nurse information). The baseline performance was also defined as the baseline mean scaled scores in some analyses (average of individual test scaled scores across the test battery).

### Cognitive decline definition

Determination of clinically relevant decline was based on previously published norms for change [22]. Standard Regression Based (SRB) change scores for each NP measure were calculated based on previously developed formulae [22]. These yielded change scores that were averaged into a mean global change score (GCS) and mean change scores for each of the seven cognitive domains. Individuals were defined as decliners if they fell below the bottom 5% cut-off point (as determined by a 95% confidence interval, one-tailed, for the mean GCS in the HIV- group). Follow-up performance was also defined as the follow-up mean scaled scores (corrected for practice effects [22]) which represents the individual test scaled scores across the test battery at follow-up.

### Statistical analyses

The proportion of decliners to non-decliners was compared between the HIV- and HIV+ groups using an exact McNemar's test. HIV- and HIV+ groups were then compared on GCS and the seven domains’ change z-scores using analysis of variance (ANOVA).

To determine how historical HAND, baseline HAND, and their interaction explained the performance at the 18th month follow-up, we conducted the following analyses. First, we investigated the relationship between historical HAND (yes/no) and HAND status at baseline (yes/no) using a chi-square analysis. Second, we conducted regression analysis with the mean scaled score at follow-up (corrected for practice effect) as an outcome variable; and three predictors: the baseline HAND performance as the baseline mean scaled score, historical HAND (yes/no), and the interaction between those two effects. The interaction term was included to assess whether having historical HAND and baseline HAND has a compounding effect on cognitive decline.

We then used the same model with the addition of the main HIV disease biomarkers as covariates to determine their relationship with cognitive decline while taking into account historical and baseline HAND (nadir CD4^+^ T cell count, current CD4^+^T cell count; standardized difference between baseline and follow-up CD4^+^T cell count; whether participants were virologically undetectable during the study period (yes/no); baseline HIV duration and baseline cART duration in months).

Analyses were conducted using JMP 11.0 version (SAS Inc.) and PASW version 18.0. The chosen study alpha level was *p <* .05 and we have provided effect sizes for all analyses.

## Results

There were no statistically significant differences between HIV-/+ participants at baseline for age, or ethnicity (Table 1). However, HIV+ participants were significantly more likely to be less educated and male. As expected, a higher proportion of HIV+ participants were impaired at baseline (Table 1). The HIV+ sample was composed of patients who were medically stable at baseline and remained stable across the duration of the study (i.e. none developed AIDS) (Table 2); 88% of HIV+ participants evidenced undetectable viral loads across the duration of the study. For the remainder, viral detection was in the form of viral blips (median 150 cp/mL, IQR:100–345). There was one single case of HIV replication beyond blip level in one participant who had to stop cART before a specific medical intervention.

**Table 1:** Baseline Demographic Variables for HIV+/- Participants.

	HIV+*M(SD)/*%	HIV-*M(SD)/*%	Statistic(*X*^*2*^*/F)*	*p*
N	96	44	-	-
Age ^a^	56.06 (7.87)	53.53 (6.50)	3.48	.06
Education (years) ^a^	13.99 (2.86)	15.23 (2.62)	6.00	.02*
Sex (% Male)	97.92%	86.26%	7.47	.01*
Ethnicity (% white)	97.92%	97.73%	0.01	.94
Impaired rate % ^b^	55.21%	15.91%%	19.03	< .001**

All information present relevant to HIV+/- participants who completed both baseline and follow-up assessment.

^a^ F-ratio, all other test statistics *X*^*2*^

^b^ Low performance rate in HIV- controls was set at ~15% to optimize specificity and sensitivity in HIV+ group (for more details see Taylor & Heaton, (2001).

* *p* < .05

** *p* < .001

**Table 2:** Means (Standard Deviations) for HIV Disease and Laboratory Characteristics at Baseline and Follow Up.

	Baseline	Follow Up	*p*
HIV duration (years)	19.18 (6.81)	-	
Nadir CD4-T cell (median cp/mL)	181.21 (125.93)	-	
Historical AIDS (%)	69.79%	No new AIDS	
Current CD4-T cell (median cp/mL)	543.03 (262.07)	638.61 (300.21)	< .001
Plasma HIV RNA (% undetectable)	97.91%	90.63%	.65
Formal historical HAND diagnosis	15.6% (15/96)	-	

Historical moderate to severe HAND had been formally diagnosed at the start of the cART era in 15/96 participants based on clinical neurological/neuropsychological standard of care at St. Vincent’s Hospital. The same standard of care has been applied to all participants at St. Vincent’s Hospital Infectious Disease Department for the duration of their HIV infection (median of 20 years). The median year of historical HAND diagnosis was 2001; one case was diagnosed in 1996 and one in 2008. The majority of patients were formally diagnosed with MND or HAD at the start of the cART era. The majority of patients were in treatment failure due to sub-optimal ART or due to resistance. For a minority, diagnosis was made during the cART while untreated, as they were unaware of their HIV diagnosis or presented late at the HIV Neurology clinics. The median interval between the formal HAND diagnosis and the start of the current study was 8 years (min = 16; max = 2).

### Rate of incident neurocognitive decline

A greater proportion of HIV+ (14.00%) participants declined as compared to HIV- cases (4.55%) but this difference was non-significant (*X*^*2*^(140) = 2.55, *p* = .11, *Φ =* .13).

**Fig 1:**
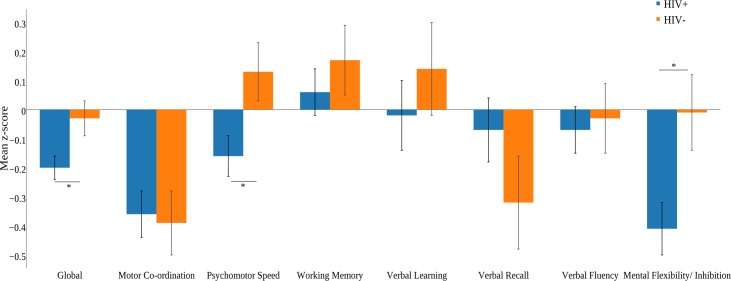
Mean global change score and cognitive domain change scores as a function of HIV status. *p*<0.05 Error bars denote standard error of the mean. Note that only one HIV+ participant improved significantly at follow-up compared to two cases in the HIV- group (*p* = 0.22).

However, the HIV+ group scored at a significantly lower level than the HIV- group on the GCS (*F*(1,138) = 4.92, *p* = .03, *d* = 0.41). HIV+ participants were also noted to score at a significantly lower level on continuous domain change scores for processing speed (*F*(1,137) = 6.06, *p* = .02, *d* = 0.46) and mental flexibility/inhibition (*F*(1,137) = 5.84, *p* = .02, *d =* 0.45). There was no significant difference between the groups on continuous change scores of motor co-ordination (*F*(1,137) = 0.05, *p* = .82, *d* = 0.04, working memory (*F(*1,137) = 0.70, *p* = .41, *d =* 0.15), verbal learning (*F*(1,137) = 0.59, *p* = .44, *d =* 0.14),verbal recall *(F*(1,137) *=* 1.73, *p* = .19, *d* = .23) and verbal fluency (*F*(1,137) = 0.08, *p* = .78, *d* = 0.05) (Fig 1).

### Cognitive determinants of decline at the group level

**Fig 2:**
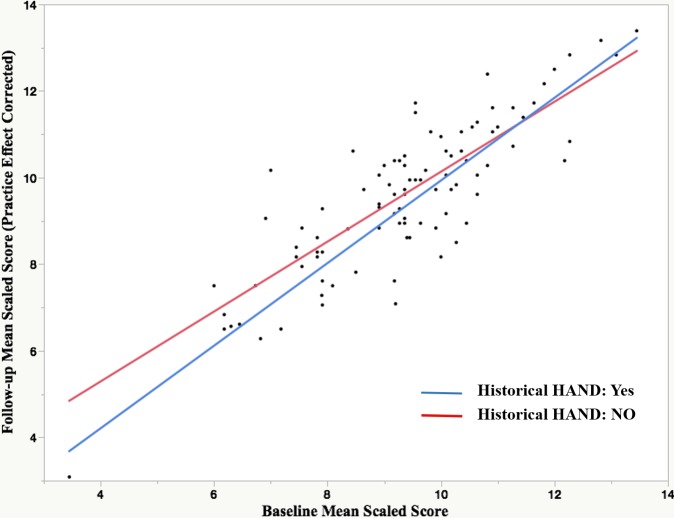
Cognitive determinants of decline. R^2^ = .73 (*p* < .0001).

Baseline HAND and historical HAND were significantly related, wherein participants who had historical HAND were more likely to also have baseline HAND (*X*^*2*^(1) = 4.40, *p* = .035). When looking at the group level using a regression analysis with the mean follow-up scaled score as the outcome variable, the driving effect on decline was whether cases had lower baseline performance on the mean scaled score (std Beta = .86; *p* < .0001). Neither past HAND status (std Beta = 0.06; *p* = .29) or the interaction between those two predictors (std Beta = -0.07; *p* = .27); (Fig 2) were significant.

### Pattern of incident cognitive decline in the HIV+ group

**Fig 3:**
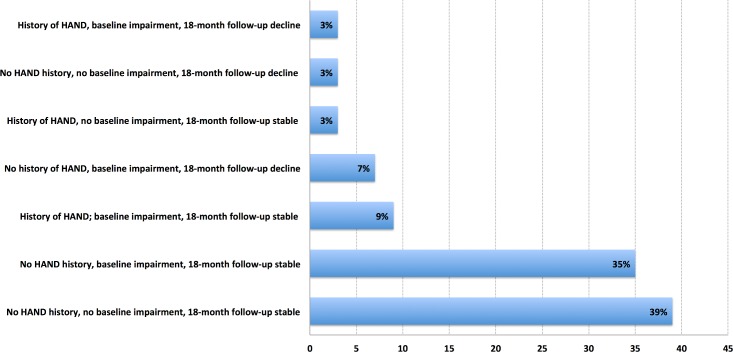
HIV+ participants’ cognitive trajectory taking into account historical HAND, baseline HAND status, and clinically significant decline.

Fig 3 illustrates the cognitive trajectory group prevalence. There were two main trajectory groups about the size of a quarter of the sample each: those who were NP-normal all the time: No HAND history, no baseline impairment, and 18-month follow-up stable performance (39%); and those who only had HAND at baseline: No HAND history, baseline impairment, 18-month follow-up stable performance (35%). This was followed by two trajectory groups who were around 8% each of the sample, and were characterised by having a history of HAND; baseline impairment, and 18-month follow-up stable performance (9%); and those with no history of HAND, baseline impairment, and 18-month follow-up decline (7%). Finally, there were three minority trajectory groups: History of HAND, no baseline impairment, and 18-month follow-up stable performance–patients who had potentially fully recovered from their initial HAND episode (3%), 6) No HAND history, no baseline impairment, 18-month follow-up decline–a minority of patients demonstrating that HAND can occur de novo, despite viral suppression (3%) 7) History of HAND, baseline impairment, 18-month follow-up decline–a minority of patients with long-term progressing HAND (3%). S3 Table presents performance on domain and global scaled scores at baseline and follow-up for HIV+ cases classified as declined, stable or improved. Only one case improved so the statistical comparisons are presented for the declined versus stable cases. Interestingly at baseline the declined cases showed weaker performance in psychomotor speed tests, attention/working memory and mental flexibility. At follow-up, there was decline across the board with most affected being, mental flexibility/cognitive inhibition, psychomotor speed, attention/working memory and Letter Fluency. Semantic Fluency was relatively spared.

### HIV disease biomarkers association with cognitive trajectories

None of the tested HIV disease biomarkers were significantly associated with decline at follow-up (Nadir CD4^+^ T cell count (*p* = .68), current CD4^+^T cell count (*p* = .16), standardized difference between baseline and follow-up CD4^+^T cell count (*p* = .72), whether participants were virologically undetectable during the study period (yes/no; *p* = .72), baseline HIV duration (*p* = .73) and baseline cART duration in months (*p* = .48).

## Discussion

There are three main findings in our study.

Firstly, in absolute terms, a higher portion of HIV+ participants showed clinically relevant decline over 18 months compared to HIV- participants. However, this result failed to meet statistical significance. Despite this, the HIV+ group showed statistically significant declining performance on the continuous global as well as domain change scores for psychomotor speed and mental flexibility/inhibitory control, relative to the HIV- group. Taken together, we interpret this discrepancy as indicating that gradual decline more commonly occurs at a slow sub-clinical rate, rather than at a rapid progressing rate in cART-treated individuals. While our study period was of 18 months, it is possible that these changes may be occurring over a longer period of time.

Secondly, the primary cognitive determinant of decline at the one follow-up occasion is performance prior to this assessment, rather than an historical episode of HAND. However, as baseline performance is in itself strongly related to any historical episode of HAND, we believe this result reflects a cascading effect. Wherein historical HAND has a mediating impact through baseline performance, rather than a direct effect on current cognitive decline. Interestingly, HIV+ cases who declined compared to those who were stable demonstrated already weaker performance at baseline in psychomotor speed, attention/working memory and mental flexibility. At follow-up they declined across the board, and more so in the weaker baseline tests, with relative sparing of Semantic Fluency.

Thirdly, while at first glance the level of neurocognitive stability is high (14% decline; one of the lowest rates of decline published). Our clinically relevant cognitive trajectories show a more complex picture; with the disease actually active to various degrees in a majority of participants. Namely those with stable baseline impairment (35%) + those with sustained long-term impairment since historical HAND, (9%) + those with baseline impairment and decline (7%), + those with incident decline at 18 months (3%) + those always progressing (3%) = 57% of the described sample.

Thirdly, none of the traditional HIV biomarkers were related to cognitive decline.

Our results are broadly consistent with previous reports which indicate that the majority of HIV+ persons on long-term cART are neurocognitively stable in reference to clinically meaningful cut-offs [9,11,13,16]. However, we also demonstrate that when taking into account historical HAND, the picture of mostly stable disease is much more nuanced. In fact, the majority of patients are undergoing slow evolution in their disease. Moreover, the moderate effect sizes detected for declines in the psychomotor speed and mental flexibility/inhibition domains further confirm this interpretation. This result was particularly striking for the psychomotor speed domain, on which the HIV- group improved at follow-up while the HIV+ group showed an overall decline (Fig 1). The specific domains affected are also of note, as decline in psychomotor speed and executive functioning have been robustly associated with ongoing HIV-related brain injury and progression to dementia [6,9,30]. The mostly sub-clinical levels of decline in domains of psychomotor and executive functioning suggests that brain injury in HIV in the cART era is primarily diffuse, probably affecting multiple connections within the striato-frontal and striato-parietal pathways [31]. Interestingly, and contrary to the study by Seider and colleagues [15], we did not find evidence for learning/memory decline, suggesting that major damage to enthorinal regions is unlikely at least within middle-aged persons. Our preference for using continuous changes scores over progression change in HAND stages, as well as clinically relevant definition of decline (*correcting for practice effect*), was key in demonstrating this more complex picture. Privileging one definition over the other, and not including practice effect corrections may yield data with poor clinical value that fails to acknowledge subtle neurocognitive change [12]. *Slow progression and subclinical deficit* may in fact represent key characteristics of the current HIV neuropathological processes in virally suppressed HIV+ persons consistent with common findings of chronic neuroinflammation rather than overt brain damage [31,32].

The fact that we do not detect any ongoing effect of traditional HIV biomarkers further indicates that there is an urgent need for elucidating the cause of HAND in virally suppressed patients with evidence of long-term CD4 recovery. Longer duration of infection [12] has also been reported a risk for cognitive decline and this was not found in this study. Potential reasons are that the duration of HIV infection was relatively homogeneous across our cohort and/or that the duration of follow-up may not have been long enough to detect any effect.

The HIV- and HIV+ group initially showed a small but significant difference in years of education corresponding to a 1.24 years gap between groups. However, this difference is not significant when grouping the educational achievement in the following categories: below high school, high school completed, and above high school (*p* = .13). This demonstrates a high proportion of overlap in the educational levels between the groups, which varies only with a year or so. Moreover, it is very unlikely that this small educational difference or any other demographic effects influenced the primary outcome of cognitive decline as the norms for change we used to quantify decline in each group were corrected for age, sex, education, Caucasian versus other ethnicity as well as baseline cognitive competence [22].

When inspecting the performance of the HIV- sample on the cognitive domain we observe relative decline in the domain of motor-coordination and verbal recall. Other domains were either stable or slightly improved. In the case of the motor-coordination domain, the relative decline lies well within the expected normal fluctuation. Indeed, using the clinically meaningful cut-off, the norms for change applied to the relevant cognitive domain [22]; 5% are expected to decline and 5% are expected to improve (90% confidence interval 2-tailed around the motor coordination change score). In the case of the HIV- group, 6.82% were classified as decliners and 2.27% were classified as improvers using this cut-off. This empirically demonstrates that while some decline is observed, the majority of performance is within the expected range. In the same domain, 11.82% of the HIV+ group was classified as decliners, and 1.05% as improvers. For verbal recall, the results are more complex. Indeed, 4.55% of the HIV- groups improved and 11.36% were classified as decliners. While the 11.36% decliner proportion was not significantly different from the expected normative 5% (Fisher’s Exact Test, one-tailed *p* = .22); this is a trend that was not observed in the HIV+ group (4.35% of the HIV- group were improvers and 5.43% were decliners). Possible explanations for this decline are twofold: normal amyloid burden variation in a large healthy middle-aged sample has been associated with verbal recall, with greater amyloid burden related to lower verbal recall [33]. In contrast, in the HIV+ group the normal amyloid variation is truncated because many individuals with potentially greater amyloid burden have already passed away [34]. Altogether, what this suggests is that the norms for change are very sensitive to even *subclinical* changes, which may or may not evolve, even in HIV- individuals.

Other limitations that should be taken into account when interpreting the findings of this study include a likely survivor bias in our cohort [7,35]. This effect may explain the lack of significant interaction between historical HAND and baseline HAND status on cognitive decline. Indeed, based on our clinical work in the hospital where this cohort was recruited, we know that the majority of persons with HAND in the pre-cART era have died. Therefore, persons from this population, who may have been more likely to decline cognitively, are now deceased. Because of this, it is possible that the observed rate of decline is an underestimate. Longer terms studies are needed both on this current population surviving from the pre-cART era as well as cohorts composed of HIV+ individuals, started on optimal cART, to reconcile how the survivor effect has impacted the current NeuroHIV longitudinal findings [18]. Finally, large collaborative international longitudinal studies are required which include large numbers of participants, reflective of the diversity of the current HIV epidemic, particularly in terms of gender and ethnicity (which the current cohort lacks).

## Conclusions

Despite long-term viral suppression, immune recovery and a likely survivor bias, our study demonstrates mostly subclinical levels of decline in psychomotor speed and executive functioning, both of which are well-established markers of HAND progression. Moreover, 57% of our cohort is undergoing some evolution of their disease over the 18-month study period. This finding challenges the notion of neurocognitive stability in virally suppressed HIV+ persons and promotes an alternate hypothesis of slow progression and subclinical deficits. Confirmation in larger and more diverse HIV cohorts will be important in shifting the focus of HIV neuropathological research towards non-overt and chronic mechanisms of brain damage.

## Supporting information

S1 Table Naturalistic longitudinal Studies of Cognition in HIV+ Individuals Stable on cART as of October 2016. HAND: HIV Associated Neurocognitive Disorder; NP: neuropsychological; BL: baseline; TMT: Trail Making Test; WAIS III: Wechsler Adult Intelligence Scale- Third Edition; WCST: Wisconsin Card Sorting Test; COWAT: Controlled Oral Word Association Test; PASAT: Paced Auditory Serial Addition Test; LNS: Letter Number Sequencing; HVLT- R: Hopkins Verbal Learning Test-Revised; BVMT-R: Brief Visuospatial Memory Test- Revised; GBTA: Group-Based Trajectory Analysis; NART: National Adult Reading Test; SDMT: Symbol Digit Modalities Test; CVLT: California Verbal Learning Test; RCFT: Rey Complex Figure Test; NR: not reported; GEE: Genrealised Estimating Equation; NS: not significant; SRB: standardised regression based; ANOVA; analysis of variance; WMS-III: Wechsler Memory Scale- 3^rd^ Edition; CVLT: California Verbal Learning Test; TOL: Tower of London; NA: CVLT-II: California Verbal Learning Test- Second Edition: RT: reaction time; RAVLT: Rey Auditory Verbal Learning Test
(PDF)

S2 Table Neuropsychological Test Battery. D: dominant hand; ND: non-dominant hand; TMT- A: Trail Making Test- Part A; WAIS-III: Wechsler Adult Intelligence Scale, 3^rd^ Edition; DKEFS- Delis Kaplan Executive Functioning System; WMS-III: Wechsler Memory Scale, 3^rd^ Edition; HVLT-R: Hopkins Verbal Learning Test- Revised; TMT- B: Trail Making Test- Part B
(PDF)

S3 Table Mean (Standard Deviation) Scaled Scores on Neuropsychological Test Battery as a Function of Declined/Stable/Improved Status at Baseline and Follow-Up. The follow-up performance is corrected for practice effect. D: dominant hand; ND: non-dominant hand; TMT- A: Trail Making Test- Part A; WAIS-III: Wechsler Adult Intelligence Scale, 3^rd^ Edition; DKEFS- Delis Kaplan Executive Functioning System; WMS-III: Wechsler Memory Scale, 3^rd^ Edition; HVLT-R: Hopkins Verbal Learning Test- Revised; TMT- B: Trail Making Test- Part B
(PDF)
