# Supplementary material for: Cognitive change trajectories in virally suppressed HIV-infected individuals indicate high prevalence of disease activity
Source: PLoS One. 2017 Mar 6;12(3):e0171887. doi: 10.1371/journal.pone.0171887 (PMC5338778; doi:10.1371/journal.pone.0171887)
Supplement: S1 Table — HAND: HIV Associated Neurocognitive Disorder; NP: neuropsychological; BL: baseline; TMT: Trail Making Test; WAIS III: Wechsler Adult Intelligence Scale- Third Edition; WCST: Wisconsin Card Sorting Test; COWAT: Controlled Oral Word Association Test; PASAT: Paced Auditory Serial Addition Test; LNS: Letter Number Sequencing; HVLT- R: Hopkins Verbal Learning Test-Revised; BVMT-R: Brief Visuospatial Memory Test- Revised; GBTA: Group-Based Trajectory Analysis; NART: National Adult Reading Test; SDMT: Symbol Digit Modalities Test; CVLT: California Verbal Learning Test; RCFT: Rey Complex Figure Test; NR: not reported; GEE: Genrealised Estimating Equation; NS: not significant; SRB: standardised regression based; ANOVA; analysis of variance; WMS-III: Wechsler Memory Scale- 3rd Edition; CVLT: California Verbal Learning Test; TOL: Tower of London; NA: CVLT-II: California Verbal Learning Test- Second Edition: RT: reaction time; RAVLT: Rey Auditory Verbal Learning Test (PDF) [file pone.0171887.s001.pdf]

Table S1: Naturalistic longitudinal Studies of Cognition in HIV+ Individuals Stable on cART as of October 2016

Year	Author	Baseline HAND Rate	Follow-up period	NP Tests	Outcome & definition of cognitive decline	Primary Outcomes
2016	Brouillette et al.	BL HAND: 46%	Every 6 months for 36 months	TMT, WAIS- III Digit Symbol Coding, WAIS-III Symbol Search, WCST, COWAT, PASAT, WAIS-III LNS, BVMT-R, HVLT-R, Grooved Pegboard	Individual Test Scores<br>GBTA<br>No correction for practice effects	15.8% declined on at least one NP test, the majority (83.8%) showed decline on only a single test<br>Attrition >10%
2006	Cysique et al.	BL HAND: 38.8%	6 , 15 & 27 months	NART, WAIS III Digit Span, TMT, SDMT, Grooved Pegboard, CVLT, RCFT, COWAT, WAIS-III Similarities	Global Score<br>Within Standard deviation based change score<br>Mixed effect regression model	6 months- 30% HIV+ individuals classified as declined, significantly higher than HIV- controls.<br>15 months: 5-13% HIV+ classified as declined<br>27 months: 5% HIV+ classified as declined<br>Attrition >10%
2007	Cole et al.	BL HAND: NR	Every 6 months over 8 years	TMT, SDMT	Individual test scores<br>GEE model<br>Linear Mixed Model	NS difference between HIV+/- groups<br>Missing cases a priori excluded

2010	Cysique et al.	BL HAND: 34%	12 months	Color Trails, WCST, COWAT, PASAT, WMS-III Spatial Span, HVLT-R, BVMT-R, Grooved Pegboard, WAIS-III Digit Span, WAIS-III Symbol Search, TMT, Stroop	Global Score<br>SRB change score<br>Repeated ANOVA	Decline in HIV+ participants (27%) significantly greater than HIV- participants (5%)<br>Attrition <10%
2014	Heaton et al.	BL HAND: 46%	Every 6 months over 16-72 months	TMT, WAIS- III Digit Symbol Coding, WAIS-III Symbol Search, WCST, COWAT, PASAT, WAIS-III, LNS, BVMT-R, HVLT-R, Grooved Pegboard	Global Score<br>SRB change score<br>Mixed effect regression model	23% HIV+ participants declined across duration of follow-up<br>Attrition >10%
2014	Seider et al.	BL HAND: NR	M=14.28 months	BVMT-R, HVLT-R	Individual test scores<br>No correction for practice effects<br>Repeated ANOVA	HIV+ participants more likely to decline on HVLT-R delay score compared to HIV- participants.<br>Attrition: NR
2015	Sheppard et al.	BL HAND: 0%	12 months	WMS-III Logical Memory, CVLT-II, WMS-III Digit Span, TMT, TOL, Grooved Pegboard	HAND diagnosis<br>No correction for pratice effects<br>Logistic Regression	HAND diagnosis at follow-up significantly higher in HIV+ participants (15.7%) as compared to impairment rate in HIV- participants (3.2%)<br>Attrition: >10%

2016	Saktor et al	BL HAND: 33%	~ every 2 years over 4 year period	TMT, Stroop, SDMT, N-back RT, RAVLT, RCFT, Grooved Pegboard	HAND diagnosis<br>No correction for pratice effects<br>Logistic Regrssion.	BL to 2 years- 70% of the HIV individuals remained at same HAND stage,15% deteriorated in HAND stage, 15% improved in HAND stage<br>2 yrs to 4 yrs- 71% of the HIV individuals remained at same HAND stage, 15% deteriorated in HAND stage, 14% improved in HAND stage<br>(e.g., MND to ANI).<br>BL to 4 years- 77% of the HIV individuals remained at same HAND stage, 13% deteriorated in HAND stage, 10% improved in HAND stage

HAND: HIV Associated Neurocognitive Disorder; NP: neuropsychological; BL: baseline; TMT: Trail Making Test; WAIS III: Wechsler Adult Intelligence Scale- Third Edition; WCST: Wisconsin Card Sorting Test; COWAT: Controlled Oral Word Association Test; PASAT: Paced Auditory Serial Addition Test; LNS: Letter Number Sequencing; HVLT- R: Hopkins Verbal Learning Test-Revised; BVMT-R: Brief Visuospatial Memory Test- Revised; GBTA: Group-Based Trajectory Analysis; NART: National Adult Reading Test; SDMT: Symbol Digit Modalities Test; CVLT: California Verbal Learning Test; RCFT: Rey Complex Figure Test; NR: not reported; GEE: Genrealised Estimating Equation; NS: not significant; SRB: standardised regression based; ANOVA; analysis of variance; WMS-III: Wechsler Memory Scale- 3rd Edition; CVLT: California Verbal Learning Test; TOL: Tower of London; NA: CVLT-II: California Verbal Learning Test- Second Edition: RT: reaction time; RAVLT: Rey Auditory Verbal Learning Test
