# Supplementary material for: Cognitive change trajectories in virally suppressed HIV-infected individuals indicate high prevalence of disease activity
Source: PLoS One. 2017 Mar 6;12(3):e0171887. doi: 10.1371/journal.pone.0171887 (PMC5338778; doi:10.1371/journal.pone.0171887)
Supplement: S2 Table — D: dominant hand; ND: non-dominant hand; TMT- A: Trail Making Test- Part A; WAIS-III: Wechsler Adult Intelligence Scale, 3rd Edition; DKEFS- Delis Kaplan Executive Functioning System; WMS-III: Wechsler Memory Scale, 3rd Edition; HVLT-R: Hopkins Verbal Learning Test- Revised; TMT- B: Trail Making Test- Part B (PDF) [file pone.0171887.s002.pdf]

Table S2: Neuropsychological Test Battery

Domains	Tasks
Motor Coordination	Grooved pegboard (DH and NDH) (score in seconds)
Psychomotor Speed	TMT-A (score in seconds)<br>WAIS-III Digit Symbol Coding (total correct/120 seconds)<br>DKEFS- Color Naming (score in seconds)
Working Memory	WAIS-III Letter-Number Sequencing (total correct sequences)<br>WMS-III- Spatial Span (total correct responses)
Verbal Learning	HVLT-R total learning (total correct Trial 1-Trial 3)
Verbal Recall	HVLT-R delayed recall (total correct)
Verbal Fluency	Letter- FAS (total correct responses)<br>Semantic- Animals (total correct responses)
Mental Flexibility/Inhibition	TMT- B (score in seconds)<br>DKEFS- Mental Control (score in seconds)

D: dominant hand; ND: non-dominant hand; TMT- A: Trail Making Test- Part A; WAIS-III: Wechsler Adult Intelligence Scale, 3rd Edition; DKEFS- Delis Kaplan Executive Functioning System; WMS-III: Wechsler Memory Scale, 3rd Edition; HVLT-R: Hopkins Verbal Learning Test- Revised; TMT- B: Trail Making Test- Part B
