# Supplementary material for: Cognitive change trajectories in virally suppressed HIV-infected individuals indicate high prevalence of disease activity
Source: PLoS One. 2017 Mar 6;12(3):e0171887. doi: 10.1371/journal.pone.0171887 (PMC5338778; doi:10.1371/journal.pone.0171887)
Supplement: S3 Table — D: dominant hand; ND: non-dominant hand; TMT- A: Trail Making Test- Part A; WAIS-III: Wechsler Adult Intelligence Scale, 3rd Edition; DKEFS- Delis Kaplan Executive Functioning System; WMS-III: Wechsler Memory Scale, 3rd Edition; HVLT-R: Hopkins Verbal Learning Test- Revised; TMT- B: Trail Making Test- Part B (PDF) [file pone.0171887.s003.pdf]

Table S3: Mean (Standard Deviation) Scaled Scores on Neuropsychological Test Battery as a Function of Declined/Stable/Improved Status at Baseline and Follow-Up

Neuropsychological Tests	Baseline			*P*	Follow-Up			*P*
	Declined	Stable	Improved	Declined vs. Stable	Declined	Stable	Improved	Declined vs. Stable
N	13	82	1		13	82	1	
Grooved Pegboard DH	7.31 (2.90)	8.35 (2.64)	9	.19	4.81 (3.19)	7.66 (2.83)	10.50	**.001**
Grooved Pegboard NDH	7.18 (3.19)	7.95 (2.49)	9	.35	6.09 (3.36)	7.54 (2.49)	9.00	.08
TMT-A	8.53 (2.30)	9.22 (2.33)	10	.32	7.12 (2.36)	8.62 (2.04)	9.50	**.02**
WAIS-III Digit Symbol Coding	7.77 (2.89)	9.56 (2.60)	10	**.02**	8.35 (2.70)	10.76 (2.65)	12.50	**.003**
DKEFS- Colour Naming	10.36 (2.62)	10.30 (2.67)	7	.94	9.70 (2.06)	10.50 (2.20)	10	.28
WAIS-III Letter Number Sequencing	9.67 (3.17)	11.38 (2.80)	9	**.05**	11.00 (3.03)	12.20 (2.45)	12	.14
WMS-III Spatial Span	8.77 (3.35)	11.01 (2.68)	12	**.008**	8.17 (2.04)	10.48 (2.70)	15	**.005**
HVLT-R Total Learning	7.08 (3.40)	8.07 (2.74)	9	.26	5.73 (2.37)	7.89 (2.81)	8	**.02**
HVLT-R Delayed Recall	6.58 (3.18)	8.12 (3.18)	11	.12	4.77 (2.15)	7.05 (3.20)	8.50	**.02**
Letter Fluency	11.31 (2.29)	10.80 (2.26)	9	.52	9.67 (2.64)	10.71 (2.27)	12	**.003**
Animal Fluency	9.38 (3.48)	10.00 (3.11)	10	.46	8.17(2.29)	10.81 (2.89)	12	.15
TMT-B	8.46 (2.15)	10.17 (2.29)	8	**.01**	6.77 (1.79)	9.23 (2.29)	11.00	**.0004**
DKEFS Inhibition	10.09 (3.39)	10.90 (2.91)	12	.40	8.40 (4.50)	11.69 (2.08)	12	**.0001**
Mean Scale Score	8.41 (1.93)	9.49 (1.64)	9.55	**.03**	7.67 (1.73)	9.81 (1.56)	11.50	**<.0001**

The follow-up performance is corrected for practice effect

D: dominant hand; ND: non-dominant hand; TMT- A: Trail Making Test- Part A; WAIS-III: Wechsler Adult Intelligence Scale, 3rd Edition; DKEFS- Delis Kaplan Executive Functioning System; WMS-III: Wechsler Memory Scale, 3rd Edition; HVLT-R: Hopkins Verbal Learning Test- Revised; TMT- B: Trail Making Test- Part B
